# Gene-associated methylation status of *ST14* as a predictor of survival and hormone receptor positivity in breast Cancer

**DOI:** 10.1186/s12885-021-08645-3

**Published:** 2021-08-21

**Authors:** Yang-Hong Dai, Ying-Fu Wang, Po-Chien Shen, Cheng-Hsiang Lo, Jen-Fu Yang, Chun-Shu Lin, Hsing-Lung Chao, Wen-Yen Huang

**Affiliations:** 1 Department of Radiation Oncology, Tri-Service General Hospital, National Defense Medical Center, No. 325, Chengong Rd., Sec. 2, Neihu, Taipei, 114 Taiwan; 2 Department of Radiation Oncology, Wan Fang Hospital, Taipei Medical University, Taipei, Taiwan; 3 Institute of Clinical Medicine, National Yang Ming Chiao Tung University, Taipei, Taiwan

**Keywords:** ST14, Matriptase, DNA methylation, Breast Cancer

## Abstract

**Background:**

Genomic profiles of specific gene sets have been established to guide personalized treatment and prognosis for patients with breast cancer (BC). However, epigenomic information has not yet been applied in a clinical setting. *ST14* encodes matriptase, a proteinase that is widely expressed in BC with reported prognostic value.

**Methods:**

In this present study, we evaluated the effect of *ST14* DNA methylation (DNAm) on overall survival (OS) of patients with BC as a representative example to promote the use of the epigenome in clinical decisions. We analyzed publicly available genomic and epigenomic data from 1361 BC patients. Methylation was characterized by the β-value from CpG probes based on sequencing with the Illumina Human 450 K platform.

**Results:**

A high mean DNAm (β > 0.6779) across 34 CpG probes for *ST14*, as the gene-associated methylation (GAM) pattern, was associated with a longer OS after adjusting age, stage, histology and molecular features in Cox model (*p* value < 0.001). A high GAM status was also associated with a higher *XBP1* expression level and higher proportion of hormone-positive BC (*p* value < 0.001). Pathway analysis revealed that altered GAM was related to matrisome-associated pathway.

**Conclusions:**

Here we show the potential role of *ST14* DNAm in BC prognosis and warrant further study.

**Supplementary Information:**

The online version contains supplementary material available at 10.1186/s12885-021-08645-3.

## Background

Progress in high-throughput gene expression profiling has now allowed for many types of genomic tests to be adopted in clinical practice for improving breast cancer (BC) classification and prognosis, such as the commercially available platforms PAM50, Mammaprint (Agenda, Huntington Beach, CA, USA), and Oncotype DX (Genomic Health, Redwood City, CA, USA) [1–3]. These tests were designed with the goal of promoting personalized treatment and management for patients with BC to ultimately improve their prognosis and survival. Although such gene-based profiling has achieved great success, recent studies have highlighted that combining gene signatures with DNA methylation (DNAm) patterns could further help to refine the molecular classification of BC [4–7].

DNAm is an epigenetic mark involving the addition of a methyl group to the cytosine pyrimidine in CpG dinucleotides [8]. Without changing the genetic sequence, DNAm alters transcription through integration of genome structures [9], and could thus serve as a useful molecular marker for gene silencing memory [10]. Indeed, BC subtypes have been associated with different methylation profiles [11]; thus, combining methylation profiling and gene expression data could help to elucidate the factors and mechanisms underlying the observed pathological and clinical heterogeneity among BC types [12]. Previous studies on epigenetic signatures of BC have mainly focused on promoter hypermethylation that leads to the silencing of tumor suppressor genes [5–7]. For example, Zhu et al. showed that methylation of the BRCA1 promoter was closely associated with decreased overall survival (OS) and disease-free survival in patients with basal-like BC [7]. Some studies revealed a high frequency of abnormal CDH1 promoter methylation in ductal breast tumors and its association with carcinogenesis [6]. However, in contrast to the wide use of the genomic tests mentioned above, methylation in only a few genes has been shown to have prognostic predictive power in BC [13]. Therefore, identification of the prognostic value of additional genes along with their methylation status is required for establishment of a complete DNAm panel.

One such candidate gene is suppressor of tumorigenicity 14 (*ST14*), which encodes matriptase, a type 2 transmembrane serine protease that plays crucial roles in physiology and cancer biology [14–16]. This protease was first discovered in BC cell lines and has been subsequently identified in various other cancer types, including ovarian, prostate, and colon cancers [17]. Overexpression of matriptase or *ST14* has also been associated with epithelial-mesenchymal transition (EMT), which in turn contributes to cancer metastasis or progression [18, 19]. In a study by Kim et al., they showed that high *ST14* expression was associated with poor survival in estrogen receptor negative patients and concluded that *ST14* is an emerging therapeutic target [20]. Accordingly, overexpression of matriptase has been shown to correlate with BC progression and a poor prognosis [21–26], whereas reduced matriptase levels could abrogate tumor progression, proliferation, and invasion in both a mouse model and BC cell lines [27, 28].

In a pan-cancer analysis, CpG methylation patterns in the promoter and gene body showed distinct correlations with gene expression levels, and different methylation patterns also influenced gene expression [29]. To date, differential expression of *ST14* according to different methylation patterns has not been investigated yet; and there were few studies addressing the role of *ST14* methylation in cancer. Existing evidence was limited to pancreatic adenocarcinoma where *ST14* is aberrantly methylated [30]. As *ST14* gene expression in BC prognosis has been reported, possible role of *ST14* methylation should be studied. Therefore, in contrast to focusing only on the promoter regions, we sought to explore the extended methylation status, including the promoter, gene body, and 3′ untranslated regions (3′ UTRs), in the DNA. We hypothesized that these gene-associated methylation (GAM) sites could cover the entire methylation information associated with a specific gene. As an example of this approach, we here highlight the impact of *ST14* methylation on OS in patients with BC, and investigate its potential as a novel prognostic biomarker.

## Methods

### Data and study design

Publicly available bioinformatics data of BC were retrieved from the Cancer Genome Atlas (TCGA), which was used as the primary dataset for obtaining the methylation profile and associated gene expression analyses. Genes associated with matriptase and EMT in BC were used to develop a classifier for the GAM status in *ST14*, which was then validated with additional datasets, GSE5364 and GSE22820. GSE75067 was used to confirm the impact of the GAM status on OS. The flowchart of the study are shown in Additional file 1: Fig. S1.

The TCGA breast carcinoma (BRCA) cohort was retrieved from Xena Browser (https://xenabrowser.net/). Initially, 1239 tumors were identified in the dataset. After excluding tumors lacking *ST14* methylation information, the final dataset included 858 tumors. Raw methylation data were based on Human Methylation 450 K (HM450K) bead arrays, and were downloaded from the Genomic Data Commons hub (https://gdc.xenahubs.net/). Following the analysis pipeline proposed by Tian et al., the raw data in IDAT format were first imported and processed with the ‘ChAMP’ package in R (version 2.22) [31]. Initially, 485,512 probes were identified. Those with a detection *p* value > 0.01 and with < 3 beads in at least 5% of samples were filtered out. Non-CpG, single nucleotide polymorphism-related probes, and multi-hit probes were further removed. Probes located in chromosomes X and Y were also excluded. The distribution of type II probes was normalized using the BMIQ function [32]. Singular value decomposition (SVD) analysis was then used to correlate the principal components with biological and technical factors. If the result of SVD analysis showed substantial technical variation, the ComBat function was used to remove the source of this variation [33]. After processing, 34 probes annotated with *ST14* remained, and each probe was characterized by its normalized β-value. We used β-value in the subsequent analyses as it corresponds approximately to the percentage of a methylated site, which is of biological effect for interpretation [34]. These probes corresponded to regions that covered the promoter, enhancer, 5′-UTR, gene body, and 3′-UTR in *ST14* (Additional file 2: Table S1). Since the mean β-value across CpG probes was shown to be associated with BC in an epigenome-wide association study [35, 36], we defined the GAM by averaging the β-values across the 34 probes. Within the dataset, the median averaged β-value was set as the cut-off screening point to determine the GAM status. The median was found to be 0.6779 (i.e., 67.79% methylation) and tumors were stratified into GAM-High and GAM-Low. Differentially methylated probes (DMPs) were identified according to the different GAM status using the limma package in R (version 3.48). Using a linear model, the CpG probes of GAM-High and GAM-Low were compared and the outputs including the average expression, logFold Change (FC), *P*-value, and t-statistic were summarized; probes with an adjusted *p*-value < 0.05 were considered to be significantly different between the groups.

Level-3, normalized RNA sequencing (RNAseq) data were downloaded from Xena Browser (https://xenabrowser.net/). Gene expression levels were quantified as reads per kilobase per million. Pearson’s correlation coefficient was used to evaluate the correlation between gene and methylation levels. The coefficients between each CpG probe and all of the matriptase- or EMT-associated genes were summed for evaluation of the overall correlation. Unsupervised hierarchical clustering was used to visualize the data clustering.

The GSE75067 dataset was based on a study assessing the association of methylation patterns with subtypes in BC [37]. The 450 K methylation data were obtained from GenomeStudio (Illumina) and converted to β-values. CpG probes with a detection *p*-value > 0.05 or number of beads < 3 were considered as missing measurements and excluded from the analysis. The bias between type I and II probes was adjusted using a peak normalization algorithm. The β-values were further smoothened by the Epanechnikov kernel function to estimate the unmethylated and methylated peaks for each chemical assay. Linear scaling was used to adjust the peaks toward 0 and 1 for the unmethylated and methylated probes, respectively. The CpG probes in *ST14* were identified and their methylation levels were adjusted with methylation median centering. This dataset included additional information about OS and HS, allowing for validation of the results derived from the TCGA cohort. Tumors with complete profiles of methylation, OS, and HS were retained for analysis, leading to a total of 144 samples.

The GSE5364 and GSE22820 datasets were used for gene expression analyses. The gene expression data were derived from the GPL96 and GPL6480 platforms, respectively. In brief, the data from GSE5364 were processed with the MAS5 algorithm and median-centering, while the data from GSE22820 were normalized with default procedures in GeneSpring 7.3.1. For genes with multiple probes, the expression of a gene was determined by geometrically averaging the probe intensities. Overall, 359 breast tumor samples were available for subsequent analyses.

### Establishment of a classifier for GAM status in *ST14*

To develop a classifier for the GAM status, genes capable of distinguishing high and low methylation levels were first included as a training set. A previous study indicated that TWIST-induced EMT triggered chromatin accessibility and alterations of DNAm in human mammary epithelial cells [38]. Moreover, EMT was shown to cause widespread genome hypo-methylation and promoter hyper-methylation variations in response to extracellular signaling [39]. Therefore, we hypothesized that genes encoding proteins associated with *ST14* and EMT might reflect the alteration of methylation levels in *ST14*. Using the keywords “ST14”, “matriptase”, “EMT”, and “epithelial-mesenchymal transition”, the related genes were searched via Kyoto Encyclopedia of Genes and Genomes (KEGG, Release 97.0), Gene Set Enrichment Analysis, and Ingenuity Pathway Analysis (Qiagen, Hilden, Germany). The literature was also searched for EMT-linked genes in BC (Additional file 3: Table S2). The combined search results were filtered and genes lacking complete profiles in the TCGA database were removed. Finally, 41 genes, inclusive of 25 *ST14*- and 16 EMT-associated genes were selected, respectively.

Least absolute shrinkage and selection operator (LASSO) was then used to reduce feature dimensionality and to select gene signatures for training. This was accomplished using the ‘glmnet’ package in R (version 4.1-1). The optimal lambda (penalty) was identified as coefficients for each shrunk gene, and only genes with non-zero coefficients were selected for training. Tumor samples were randomly assigned as training and testing datasets at a 7:3 ratio, and each sample was labeled according to GAM-High or GAM-Low. The following six algorithms were employed to assess the accuracy of the classifiers: Logistic regression (LR), K-nearest neighbor, support vector classifier 1 (SVC1, using a linear kernel), SVC2 (using a radial basis function kernel), Gaussian naive Bayes, decision tree (DT), and random forest. The performance of the classifiers was evaluated by the Receiver operating characteristic (ROC) analysis with the area under curve (AUC) value and 10-fold cross validation. The classifier with the best accuracy and performance was chosen for GAM status prediction in the GSE5364 and GSE22820 datasets, which was partially validated by the *ST14* expression in each dataset. The preparation of training, testing datasets, preprocessing, model evaluation, and prediction was conducted using the ‘scikit-learn’ package (version 0.23.0) in Python (version 3.7.4).

### Differential gene expression analysis

BioJupies, which applies limma and a geometrical approach (characteristic direction), was used to identify differentially expressed genes (DEGs) [40–42]. The preprocessed expression data for the 41 genes from the TCGA, GSE5364, and GSE22820 datasets were uploaded. Individual samples in each dataset were divided into GAM-High and GAM-Low. Significant DEGs with an adjusted *p*-value < 0.05 were identified and visualized with a Volcano plot.

### Gene ontology pathway analysis and network construction

In TCGA and GSE75067, DEGs were also derived for GAM status across the whole transcriptome. DEGs were pooled and Metascape (http://www.metascape.org) was used to assess the overexpression of Gene Ontology categories in biological networks and KEGG pathways.

### Association of hormone status (HS) positivity and GAM status

Several studies have reported a correlation between CpG methylation and HS in BC [43, 44]. Therefore, we assessed this hypothesis to determine whether HS positivity is associated with the GAM status and CpG methylation in *ST14* using Chi-square test. Tumors with complete information on HS, i.e., estrogen receptor (ER) or progesterone receptor (PR) expression, from TCGA and GSE75067 were observed for this association, including a total of 889 eligible samples (TCGA, *n* = 745; GSE75067, *n* = 144). CpG probes with potential to classify the HS were identified with a feature selection method combining LASSO and the recursive feature elimination (RFE) algorithm (‘caret’ package in R, version 6.0–88). For the potential probes, their median β-values were set as the cut points for division into high and low methylation level to observe the distribution of HS.

### Statistical analysis

Chi-Square test and the Wilcoxon rank-sum test were used for analyses of categorical and continuous variables, respectively; *p* <  0.05 was considered statistically significant. Kaplan-Meier (KM) analysis with the log-rank test was used to evaluate the distinguishing effect of GAM status on OS. Multivariate Cox model analysis was used to evaluate the impact of GAM on survival. All statistical analyses were conducted in R software (version 3.6.1).

## Results

### Defining the GAM status of *ST14* and developing a classifier for GAM status

**Fig. 1:**
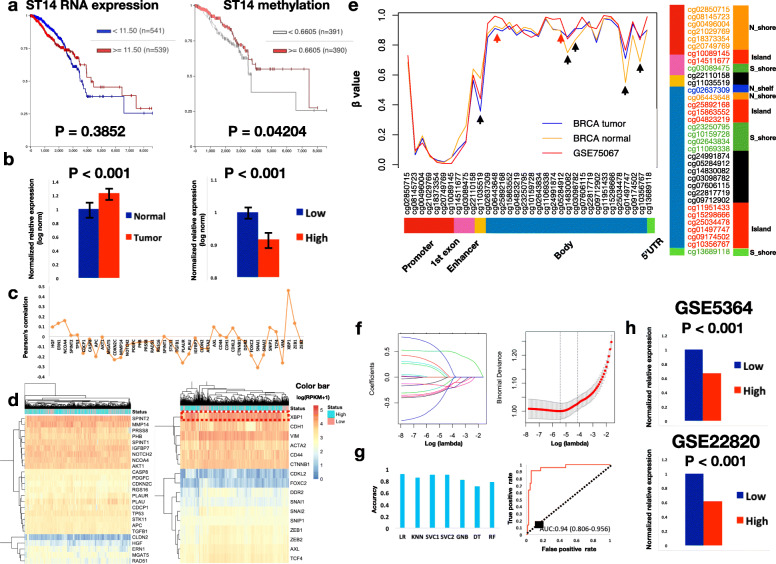
Characterization of *ST14* methylation and establishment of a classifier for gene-associated methylation (GAM) status. **a** Kaplan-Meier curve for overall survival divided into two groups based on the median *ST14* expression level (left panel, log-rank *p* = 0.382) and methylation level (right panel, log-rank *p* = 0.042) in the TCGA BRCA cohort. The median split for gene expression was based on the RNAseq data (defined as log2(RPKM+ 1)) (11.5). Average β-values across the 40 CpG probes was calculated and their median value across all samples is 0.6605. RNAseq, RNA sequencing; RPKM, reads per kilobase per million mapped reads. **b** Difference in *ST14* gene expression levels between normal and primary tumors in the TCGA BRCA cohort (left panel, Wilcoxon’s *p* < 0.001). The difference of *ST14* expression level between high and low methylation status groups (defined by the median methylation value) is shown on the right panel (Wilcoxon’s *p* < 0.001). Error bars show the standard deviation. **c** Methylation profile (34 CpG probes) of *ST14* for TCGA tumor (blue) TCGA normal tissue (orange) and GSE75067 (red). The corresponding cgi probes and features are shown in the lower and right panels. Red arrows indicate regions with large differences (Average β-values > 0.125) between TCGA and GSE75067 tumors; black arrows indicate the regions with large differences between TCGA tumor and normal samples (**d**) Pearson’s correlation coefficients between genes involved in matriptase-associated or epithelial mesenchymal transition (EMT)-associated pathways and the GAM in TCGA cohort. The highest positive correlation was noted for *XBP1*. **e** Unsupervised hierarchical clustering for matriptase-associated (left panel) and EMT-associated genes (right panel) in the TCGA cohort with GAM status. The gene expression level is based on log2 (RPKM+ 1) transformation of RNAseq data, with the color bar shown in the upper-right corner: high GAM status is in cyan, and low GAM status is in pink. **f** Identification of the optimal lambda value for the least absolute shrinkage and selection operator (LASSO). The left panel depicts the shrinkage of coefficients and the right panel shows the binomial deviance during shrinkage. The optimal lambda was 0.003970773. **g** Assessment of classifier accuracy (left panel). LR, logistic regression; KNN, K-nearest neighbor; SVC1, support vector classifier 1 (using a linear kernel); SVC2, support vector classifier 2 (using a radial basis function kernel); GNB, Gaussian naive Bayes; DT, decision tree; RF, random forest. The highest accuracy was obtained with LR (accuracy = 91.31%). Receiver operating curve for LR with the area under curve value and 95% confidence interval shown in the lower part. **h** Normalized gene expression levels of *ST14* between high and low GAM status groups for GSE5364 (upper panel, *p* < 0.001) and GSE22820 (lower panel, *p* < 0.001). The GAM status was predicted using the classifier constructed by LR

In the KM analysis in Xena browser, there was no association between *ST14* expression and OS (*p* = 0.3852, Fig. 1a), whereas patients with a higher methylation status (average β-value > 0.6605) in *ST14* had significantly better OS (*p* = 0.04014). In addition, a higher *ST14* expression level was identified in breast tumors as compared with that in normal tissues (Wilcoxon’s *p* value < 0.001, Fig. 1b) and in tumors with a lower methylation status (Wilcoxon’s *p* value < 0.001). Taken together, these results suggested that the methylation status of *ST14* was related to its expression and might provide improved survival prediction than gene expression itself.

To confirm this hypothesis, GSE75067 dataset, based on a study assessing the association of methylation patterns with subtypes in BC was used. After removing missing data, 144 samples were included in our analysis. After pre-processing, 34 probes remained and the distribution of β-values across the 34 probes in the TCGA (primary tumors and normal tissues) and GSE75067 datasets showed extremely similar patterns, except for some CpG sites in the gene body (mean differences > 0.125, Wilcoxon’s *p* value < 0.001)(Fig. 1c). β-value is the estimate of methylation level by using the ratio of intensities between methylated and unmethylated probes, with 0 being unmethylated and 1 being fully methylated. Using a median averaged β-value (0.6779) across the 34 probes, GAM was derived and stratified into GAM-High and GAM-Low. We then evaluated the predictive potential of the 41 *ST14*- and EMT-associated genes for GAM status. Using Pearson’s correlation, we first observed that XBP1 had the strongest positive correlation with GAM in the TCGA cohort (Fig. 1d, Pearson’s coefficient = 0.458, *p* = 2.58e-5). This higher correlation was also observed in the hierarchical clustering, in which the *XBP1* expression level showed strong clustering with GAM status (Fig. 1e).

Using LASSO, genes with non-zero coefficients were identified (Fig. 1f; Additional file 4: Table S3), which were *ACTA2*, *APC*, *AXL*, *IGFBP7*, *MMP14*, *PLAU*, *PLAUR*, *SNAI1*, *SNAI2*, *SPINT2*, *XBP1*, and *ZEB2*. These 12 genes were then used as the gene signature for training in the TCGA dataset. Among the algorithms applied, LR had the highest accuracy in classification (accuracy = 91.31%), followed by SVC1 (accuracy = 90.25%) and SVC2 (accuracy = 90.25%), and DT had the lowest accuracy (accuracy = 70.65%) (Fig. 1g). ROC analysis showed a high AUC (0.94, 95% confidence interval = 0.806–0.956) for LR. Therefore, LR was chosen as the classifier for GAM status in our study, which was then used for GAM status prediction in the GSE5364 and GSE22820 datasets. These two datasets, based on GPL96 and GPL6480 platforms respectively, contained genomic data for BC and were used for validation. Both validating datasets showed significantly lower *ST14* expression levels in GAM-High, similar to that observed in the TCGA cohort (Fig. 1h), suggesting the feasibility of our classifier.

**Fig. 2:**
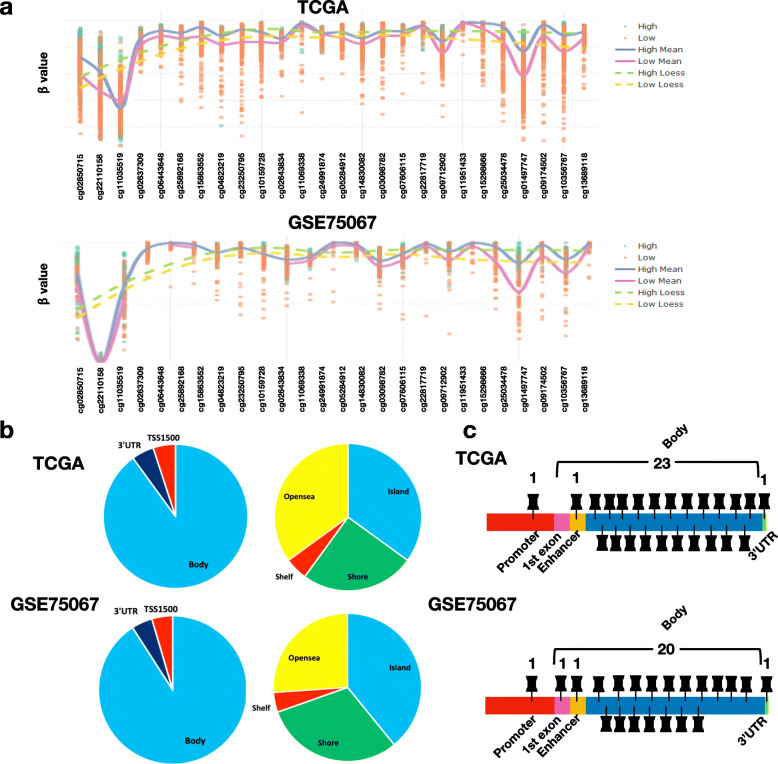
Differential methylation analysis between tumor samples with high and low gene-associated methylation (GAM) status. **a** Distribution of mean β-values for the differentially methylated probes (DMPs) between high and low GAM status groups in the TCGA and GSE75067 databases (solid line). The dashed line indicates the local regression line fitted for high or low GAM status groups. The purple and blue line connects the mean β-value for each probe in the high and low GAM status group, respectively. All probes shown here are significantly different between the high and low GAM status groups, based on a linear model for microarray data. **b** Pie charts and diagrams for visualization of the DMPs annotated with genetic features and CpG features for TCGA (upper panel) and GSE75067 (lower panel) datasets. **c** Locations of DMPs in *ST14* are indicated

DMPs between the GAM-High and GAM-Low were identified and are summarized in Additional file 5, 6: Table S4–5. Probes identified to be the DMPs were similar between the TCGA and GSE75067 datasets (Fig. 2a and b). Moreover, 23 and 20 DMPs were found in the gene body in the two datasets, accounting for 88.5 and 83.3% of the overall DMPs, respectively (Fig. 2c). The CpG probe with the greatest difference between groups was identified as cg01497747 in both datasets, with a logFC of 0.161 and 0.235 for TCGA and GSE75067, respectively.

### Target gene for the GAM status of *ST14*

**Fig. 3:**
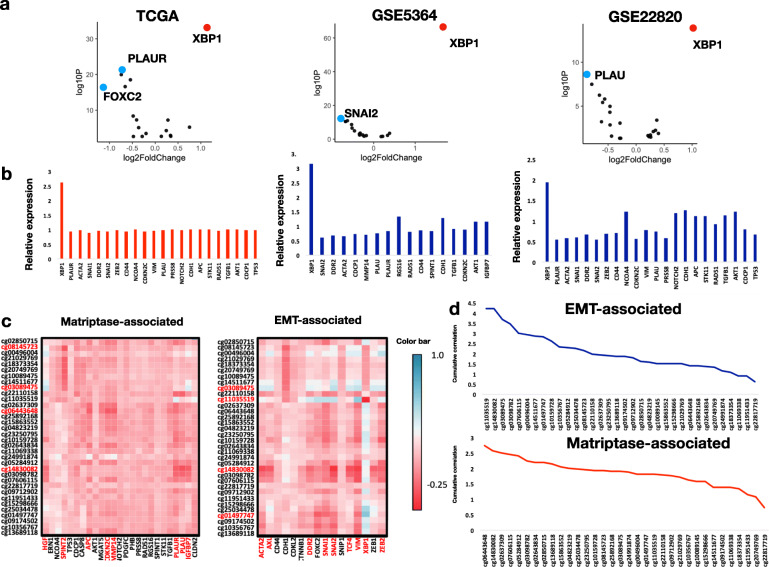
Differential gene expression and correlation analyses. **a** Volcano plot analyses for genes involved in matriptase-associated and epithelial-mesenchymal transition (EMT)-associated pathways in TCGA, GSE5364, and GSE22820 datasets. In these three datasets, *XBP1* was significantly up-regulated with a high -log10P value in the high GAM status group (red). Conversely, *PLAUR*, *FOXC2*, and *SNAI2* were significantly down-regulated with a high -log10P (blue). **b** Relative expression levels of the differentially expressed genes in the three datasets, with low GAM status as the referencing condition. **c** Correlation heatmap between CpG probes, matriptase-associated genes (left panel), and EMT-associated genes (right panel). The color bar for Pearson’s correlation coefficients is shown on the right. Genes and probes with higher correlation (abstract coefficient > 3) are colored in red. **d** Overall correlation between CpG probes, matriptase-associated genes (left panel), and EMT-associated genes (right panel)

Focusing on the aforementioned 41 genes, differential gene expression analysis between GAM-High and -Low identified 18, 18, and 22 DEGs with significantly altered expression in the TCGA, GSE5364, and GSE22820 datasets, respectively (Additional file 7, 8 and 9: Table S6-8S and Fig. 3a). In these three datasets, *XBP1* was found to be up-regulated in the GAM-High, with the largest FC of 1.66 in the GSE5364 dataset. In the TCGA cohort, *PLAUR* was down-regulated in the GAM-High (logFC = − 0.747, −log10p = 21.61), which was also observed in GSE22820. The relative expression levels of these DEGs between the two GAM status groups are shown in Fig. 3b, supporting that the *XBP1* expression level was significantly higher in GAM-High (Wilcoxon’s *p* value < 0.001).

Among the correlations between the CpG probes and the expression levels of the 41 genes, the highest abstract correlation was observed between cg11035519 and *XBP1* in the TCGA cohort (Pearson’s coefficient = − 0.5919; Fig. 3c). Of note, cg11035519 was annotated as an enhancer in *ST14*. Furthermore, we identified that cg11035519 and cg14830082 had the highest cumulative correlations with EMT-associated genes (overall Pearson’s coefficient = 4.2, Fig. 3d), implying the presence of a regulatory site between the enhancer and EMT-associated genes. By contrast, there was a relatively lower correlation observed between the CpG probes and the matriptase-associated genes, with cg06443648 showing the highest cumulative correlation (overall Pearson’s coefficient = 2.7). Further correlation analysis in the TCGA dataset showed a moderately positive correlation between cg11035519 methylation and expression levels of *FOXC1*, *FAM171A*, *RGMA*, *SFRP1*, *CHST3*, and *PTX3* (all coefficients > 0.6, *P* <  0.05, Additional file 10: Table S9).

### Association of *ST14* GAM status with molecular features in BC

**Fig. 4:**
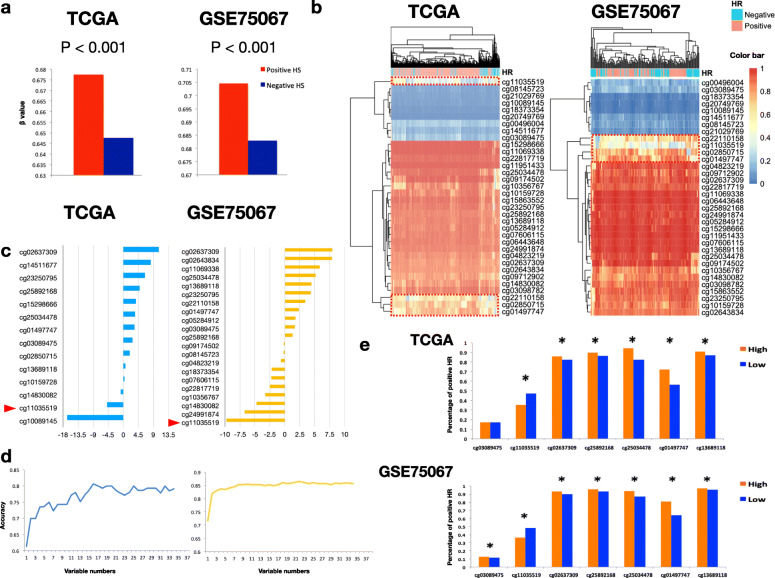
Association of *ST14* methylation with hormone receptor status (HS) positivity in TCGA and GSE75067. **a** Mean GAM between positive and negative HS. Both datasets showed a significant difference of GAM, with a higher GAM observed in HS-positive breast cancers (*p* < 0.001). **b** Unsupervised hierarchical clustering for CpG probes and the HS. The β-value is indicated by the color bar in the upper-right corner; a positive HS is in pink and negative HS is in cyan. **c** Vertical bar plots for CpG probes with non-zero least absolute shrinkage and selection operator (LASSO) coefficients for TCGA (left panel) and GSE75067 (right panel). **d** Plot of recursive feature elimination (RFE) classification accuracy for the HS with probe numbers for TCGA (left panel) and GSE75067 (right panel). The selected probe set showed the highest accuracy. In TCGA, 16 CpG probes were selected, whereas 22 probes were selected in GSE75067. **e** CpG probes selected by LASSO and RFE in both datasets. The methylation status is defined by the median split for each probe. The percentage of positive HS is plotted against each selected probe with high and low methylation status. Significance of the difference is indicated by a star (Chi-square test *p* < 0.05)

In the TCGA and GSE75067 datasets, the distribution of PAM50 molecular features was significantly different between GAM-High and GAM-Low (Table 1). Furthermore, hormone receptor status (HS) was also significantly different between the two statuses (Table 2), in which a GAM-High was associated with a positive HS. Moreover, GAM was also significantly higher for the positive HS samples (Fig. 4a). Unsupervised hierarchical clustering showed higher clustering of cg11035519 with the HS in the TCGA and GSE75067 cohorts, with higher methylation corresponding to negative HS (Fig. 4b). Using LASSO to select potential CpG probes, we observed that cg10089145 had the most negative coefficient (− 16.7349), followed by cg11035519 (− 4.8834) (Fig. 4c) in TCGA. The negative influence of cg11035519 was confirmed in the GSE75067 dataset (coefficient = − 9.77947). Probes with a non-zero coefficient were further eliminated with RFE to find the most predictive probes for HS (Fig. 4d). Combining LASSO and RFE, seven CpG probes were identified, which were cg03089475, cg11035519, cg02637309, cg25892168, cg01497747, and cg13689118. These seven probes corresponded to the enhancer, gene body, and the 3′-UTR in *ST14*. Except for the probe annotated to the enhancer (cg11035519), the other CpG probes with a high β-value harbored higher portion of samples with positive HS in both TCGA and GSE75067 (*p* <  0.001, Fig. 4e).

**Table 1:** Association of GAM status with PAM50 molecular feature in TCGA and GSE75067

PAM50	Overall	Low (β ≤ 0.6779)	High (β > 0.6779)	***P***
**TCGA**				
Normal	17	12	5	< 0.001
Luminal A	277	36	241	
Luminal B	126	23	103	
Her2	31	20	11	
Basal	86	60	26	
**GSE75067**				
Normal	11	5	6	0.002
Luminal A	34	10	24	
Luminal B	26	10	16	
Her2	26	15	11	
Basal	38	20	18	

*TCGA* the Cancer Genome Atlas

**Table 2:** Distribution of HS of TCGA and GSE breast cancer patients grouped by GAM status of *ST14*

	Overall	GAM status		*P*
		Low (β ≤ 0.6779)	High (β > 0.6779)	
TCGA				
Hormone receptor		*n* = 260	*n* = 485	
Positive	584	144	440	< 0.001
Negative	161	116	45	
GSE75067				
Hormone receptor		*n* = 40	*n* = 104	
Positive	85	8	77	< 0.001
Negative	59	32	27	

*HS* Hormone status, *GAM* Gene-associated methylation

### Survival analysis

**Fig. 5:**
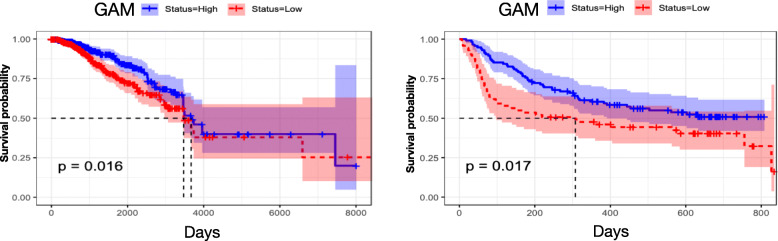
Kaplan-Meier (KM) survival analysis for overall survival (OS) in TCGA and GSE75067. The samples in the two datasets were divided into high and low gene-associated methylation (GAM) status groups based on the study-defined β-value (0.6779). The KM plots also show areas with 95% confidence intervals

The KM plot demonstrated a significant survival difference between the GAM-High and GAM-Low in both TCGA (log-rank test *p* = 0.016) and GSE75067 (log-rank test *p* = 0.018) (Fig. 5). With longer follow-up in the TCGA cohort, GAM-High patients had a median survival (MS) of 122.3 months, compared with 115.7 months in the low GAM status group. Intriguingly, a drop in survival occurred within 3 months for GAM-Low patients in GSE75067, and these patients showed a 15.8-month shorter MS than those with GAM-High (MS: 5.3 vs 21.1 months). This pattern was not observed in the TCGA dataset. These findings suggested that GAM could help risk stratification in BC. Further, GAM status remained the only significant factor after adjusting age, pathological stage, histologic type and PAM50 subtypes in the multivariate Cox model (Table 3).

**Table 3:** Multivariate analysis of covariates for survival in breast cancer

Covariates	HR	95% CI	***P***
**TCGA**			
GAM status (ref: GAM-Low)	0.16	0.07–0.37	< 0.001
Age (ref: <= 50)	2.19	1.11–4.35	0.023
Pathological stage (ref: stage I)	2.79	0.86–6.05	0.09
Histologic type (ref: IDC)	0.52	1.11–4.35	0.13
PAM50 (ref: normal)			
Luminal A	0.51	0.10–2.56	0.41
Luminal B	0.74	0.15–3.71	0.71
Basal	0.28	0.05–1.39	0.11
Her2	0.47	0.07–3.06	0.42
**GSE75067**			
GAM status (ref: GAM-Low)	0.39	0.21–0.74	0.003
Age (ref: <= 50)	2.03	1.25–3.30	0.004
Histologic type (ref: IDC)	0.61	0.29–1.26	0.18
PAM50 (ref: normal)			
Luminal A	1.99	0.43–9.27	0.37
Luminal B	4.73	1.07–20.96	0.04
Basal	4.64	1.05–20.59	0.04
Her2	7.75	1.77–34.1	0.006

*HR* hazard ratio, *CI* confidence interval, *GAM* gene associated methylation, *IDC* invasive ductal carcinoma

### Gene ontology analysis of differential genes for GAM status

**Fig. 6:**
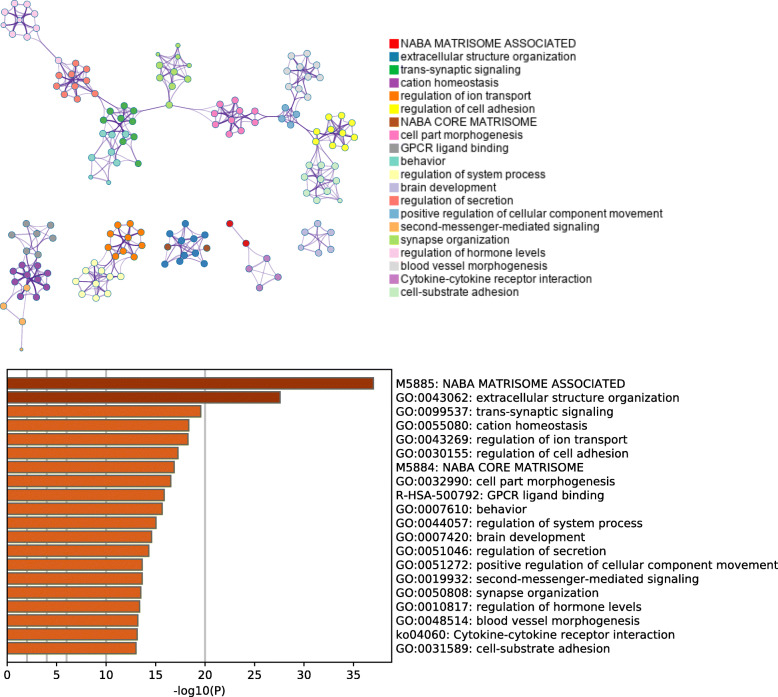
Gene Ontology and pathway enrichment analysis of genes involved in methylation of *ST14*

The genes differentially expressed between different GAM statuses were analyzed using Gene Ontology and KEGG analysis. Two thousand nine hundred ninety-eight DEGs were identified (Additional file 11). We found the most enriched pathways are ‘NABA MATRISOME ASSOCIATED’ and ‘extracellular structure organization’ (−log10p > 20) (Fig. 6). This finding suggested genes involved in regulation of extracellular microenvironment could alter the GAM status in *ST14*.

## Discussion

Identification of new biomarkers for BC can help to optimize the treatment of patients with varying disease spectra. Since cancer phenotypes are determined by both epigenetic modifications and genetic aberrations, investigation of epigenetic variations and integration of these data can improve risk stratification and cancer prognosis. Here, we found that the GAM status in *ST14* was associated with OS in patients with BC. This association was also observed for progression-free survival in the TCGA BRCA primary tumor cohort (Additional file 12: Fig. S2). These observations suggest GAM can potentially serve as a biomarker for OS in patients with BC. Several studies reported that a lower average methylation level across the epigenome in blood DNA was associated with a higher risk of BC [35, 36]. Alternatively, in the present study, lower tissue *ST14*-associated methylation (i.e., GAM status) was associated with a higher risk of death, providing a surrogate for risk evaluation in BC. Furthermore, a higher *ST14* expression level was significantly associated with a low GAM status in the training TCGA and the two validation cohorts. This implies the GAM status could alter the transcription efficiency and that a higher methylation level inhibits gene expression, suggesting that DNAm is more generalized than gene expression.

According to a recent review from Smith et al., the paradigm of promoter DNA methylation as a transcriptional silencing mechanism does not always hold true [45], and hypermethylation-induced transcriptional activation has been documented in a range of cellular changes, including development, malignancy, and metastatic disease. Under some circumstances, methylation of gene promoters facilitates context-dependent transcriptional activation across a range of biological settings. Therefore, gene methylation is regulated differently from its expression and could serve as biomarkers for certain endpoints. One well known example is the promoter methylation of MGMT, not its gene expression correlates well to the efficacy of Temozolomide in glioblastoma multiforme [46]. Although promoter methylation is a major mechanism of gene silencing, additional factors may affect the correlation between MGMT methylation, expression, and patient outcome. Alternative mechanisms, such as post-transcriptional modulation of MGMT by miRNAs or the association of MGMT methylation with IDH mutation or the glioma CpG island methylator phenotype, may explain these inconsistent correlations and different outcomes. Moreover, using DNA methylation as a predictor has a few advantages over other biomarkers. For instance, DNA methylation had a higher stability both in vivo and ex vivo, the requirement of a smaller amount of specimens to obtain enough DNA for analyzing methylation, and higher accuracy. Some study suggested that combinations of DNA methylation as predictors may yield higher sensitivity and specificity than individual DNA methylation. As shown in Fig. 1b in our manuscript, hypermethylation of *ST14* is associated with its lower expression. Therefore, the direct impact could be partly explained by the paradigm repressive role on its gene expression. Since different regulatory mechanisms exist and therefore varying observations in gene expression and methylation detected, survival impact could be different between gene expression and methylation assays.

Yang et al. reported a positive relationship between DNAm in the gene body and gene expression [47]. Another study in hepatocellular carcinoma supported this idea and identified that CpG hypermethylation in the gene body region was linked to the upregulated expression of several oncogenes [48]. Similarly, in the present study, hypermethylation in the gene body region was correlated with a higher *ST14* gene expression level in tumor tissues compared with that in normal tissues in the TCGA BRCA cohort (Additional file 13, 14: Fig. S3–4). By contrast, within tumor samples, DMPs with higher methylation in the gene body were found to be linked to a high GAM status, which was in turn associated with a low *ST14* expression level. This finding suggests a threshold methylation level in tumor samples that could further modify the transcription efficiency. *XBP1* was previously identified as a candidate oncogene that is induced in various cancer types. After being cleaved by inositol-requiring enzyme 1ɑ (IRE1), a functional spliced variant of *XBP1* mRNA (*XBP1S*) is formed, which plays a role in the unfolded protein response and autophagy [49–52]. Accumulating evidence supports a direct role of *XBP1* in tumor invasion and metastasis [53, 54]. Moreover, *XBP1* was reported to be activated in triple-negative BC, contributing to tumorigenesis through the HIF-1ɑ pathway [55]. In addition, the level of *XBP1S* expression was higher in basal-like BC cell lines, supporting the adverse effect of this variant [56]. By contrast, levels of unspliced *XBP1* (*XBP1U*) mRNA correlated with *ESR1* mRNA levels in luminal-type BC, and higher levels of *XBP1U* mRNA were associated with better survival in patients with BC [57]. Furthermore, higher ratios of *XBP1S/XBP1U* mRNA level were associated with poor survival. Collectively, these findings suggest that *XBP1* isoforms have distinct functions and that their expression is cell type-specific. Most of the tumor samples in the TGCA cohort were of the ER-positive luminal subtypes (Additional file 15: Fig. S5), implying a higher portion of *XBP1U* in this cohort. The high correlation coefficient between *XBP1* and *ESR1* supports this hypothesis (Pearson’s correlation = 0.7754, *p* = 1.58e-10). In the differential expression analysis, *XBP1* was significantly up-regulated in the GAM-High, which corresponded to a more positive HS and better OS. As such, higher *ST14* methylation might be associated with higher *XBP1U* expression, suggesting a novel link between *XBP1* and DNAm.

Among the DMPs identified, cg11035519 was found to be the most negatively correlated with *XBP1* expression and had the highest overall correlation with EMT-associated genes. cg11035519 was annotated to an enhancer in *ST14*, and is located in the local minimum after the first exon. In the reference genome hg19, this probe is situated in ch11:130,051457–130,051459, which is the intron region of *ST14*. These findings suggest the possible existence of enhancer RNA that could affect the transcription of downstream oncogenes [58]. Of note, genes with mRNA levels positively correlated with the levels of cg11035519 methylation were reported to be induced in basal-like BC [59, 60], supporting a role in downstream activation, and a negative correlation between *XBP1U* and the basal-like histology. Moreover, we found a negative correlation between cg11035519 and the HS, which was not found for the other predictive CpG probes. Taken together, these results suggest that cg11035519 might be independently regulated in *ST14*.

Previous studies have shown that ER-positive BC with a primary origin displayed more hypermethylated loci and a higher overall DNAm than ER-negative or secondary ER-positive breast tumors [61–64]. Moreover, Fackler and colleagues used the HM27 platform to analyze samples from 103 patients with BC, which identified a classifier for HS with 40 CpG probes [62]. Another study demonstrated that hypermethylation of the promoters of several genes could strongly predict the HS in BC [44]. Taken together, these findings suggest that DNAm in a specific set of genes could reflect the HS in BC and might be used to predict a clinical benefit with hormone therapy. Although DNAm in *ST14* has never been reported to correlate with the HS, several of the CpG probes identified in our study did show strong clustering with the HS and high classifying power. This suggests that the HS is also an epigenomic event, which in turn supports the finding of a different overall DNAm profile between ER-positive and ER-negative tumors. Indeed, in our study, GAM-High tumors were characterized by a higher percentage of positive HS. From a clinical viewpoint, ER/PR-positive BC is associated with a better outcome, supporting our survival findings.

Our study had several limitations. First, the GAM defined in our study could be further optimized via advanced bioinformatic methods, accounting only for probes that are of biological meaning and predictive power. Second, only DNAm in *ST14* was investigated in this study. There might be other potential genes with prognostic power in terms of GAM. Further study could be done to elucidate the interaction network of GAM in BC oncogenes. Third, the validation datasets are limited. Future establishment of validation datasets to confirm the role of GAM is required.

## Conclusions

Our study identified that BC with a low GAM status in *ST14* was associated with poorer survival and a negative HS. In the era of personalized medicine, these findings could help refine the epigenomic panel for BC classification and prognostic evaluation. Before clinical use, its epigenetic modulation, relationship with EMT-associated genes, especially *XBP1*, and feasibility as a clinical biomarker require further confirmation. Nevertheless, these results still provide a new perspective on the role of matriptase from the point of epigenetic regulation in BC.

## Supplementary Information

**Additional file 1: Figure S1.** Study flow chart.

**Additional file 2: Table S1.** Detailed information of 34 probes annotated with *ST14.*

**Additional file 3: Table S2.** EMT biomarkers in breast cancer.

**Additional file 4: Table S3.** Coefficient of genes derived from LASSO.

**Additional file 5: Table S4.** DMPs for TCGA between high and low GAM groups.

**Additional file 6: Table S5.** DMPs for GSE75067 between high and low GAM groups.

**Additional file 7: Table S6.** Differential gene expression results for TCGA BRCA cohort.

**Additional file 8: Table S7.** Differential gene expression results for GSE5364.

**Additional file 9: Table S8.** Differential gene expression results for GSE22820.

**Additional file 10: Table S9.** Correlation between cg11035519 and gene expression of *FOXC1*, *FAM171A*, *RGMA*, *SFRP1*, *CHST3*, and *PTX3.*

**Additional file 11.** DEGs between high and low GAM groups.

**Additional file 12: Figure S2.** Progression-free survival in BRCA cohort.

**Additional file 13: Figure S3.** Snapshot of CpG beta value distribution between normal and tumor samples from Xena browser.

**Additional file 14: Figure S4.** DMPs between normal and tumor tissues in TCGA BRCA cohort.

**Additional file 15: Figure S5.** Snapshot of the percentage of hormone status for TCGA BRCA cohort.

## Data Availability

The data that supports the findings of this study are available from TCGA (https://xenabrowser.net/) and gene expression omnibus (Accession number: GSE5364, GSE22820 and GSE75067). Further details and other data are available from the corresponding author upon request.

## Abbreviations

AUC: Area under curve
BC: Breast cancer
BRCA: Breast carcinoma
DEG: Differentially expressed gene
DMP: Differentially methylated probe
DNAm: DNA methylation
EMT: Epithelial-mesenchymal transition
ER: Estrogen receptor
FC: Fold change
GAM: Gene-associated methylation
HM450K: Human Methylation 450 K
HS: Hormone status
IRE1: Inositol-requiring enzyme 1ɑ
KEGG: Kyoto Encyclopedia of Genes and Genomes
KM: Kaplan-Meier
LASSO: Least absolute shrinkage and selection operator
LR: Logistic regression
MS: Median survival
OS: Overall survival
3′ UTR: 3′ untranscribed region
PR: Progesterone receptor
RFE: Recursive feature elimination
RNAseq: RNA sequencing
ROC: Receiver operation characteristic
ST14: Suppressor of tumorigenicity 14
SVC: Support vector classifier
SVD: Singular value decomposition
TCGA: The Cancer Genome Atlas
*XBP1S*: Spliced variant of *XBP1* mRNA
*XBP1U*: Unspliced *XBP1*
