# Supplementary figures and images for: Gene-associated methylation status of ST14 as a predictor of survival and hormone receptor positivity in breast Cancer
Source: BMC Cancer. 2021 Aug 21;21:945. doi: 10.1186/s12885-021-08645-3 (PMC8380334; doi:10.1186/s12885-021-08645-3)

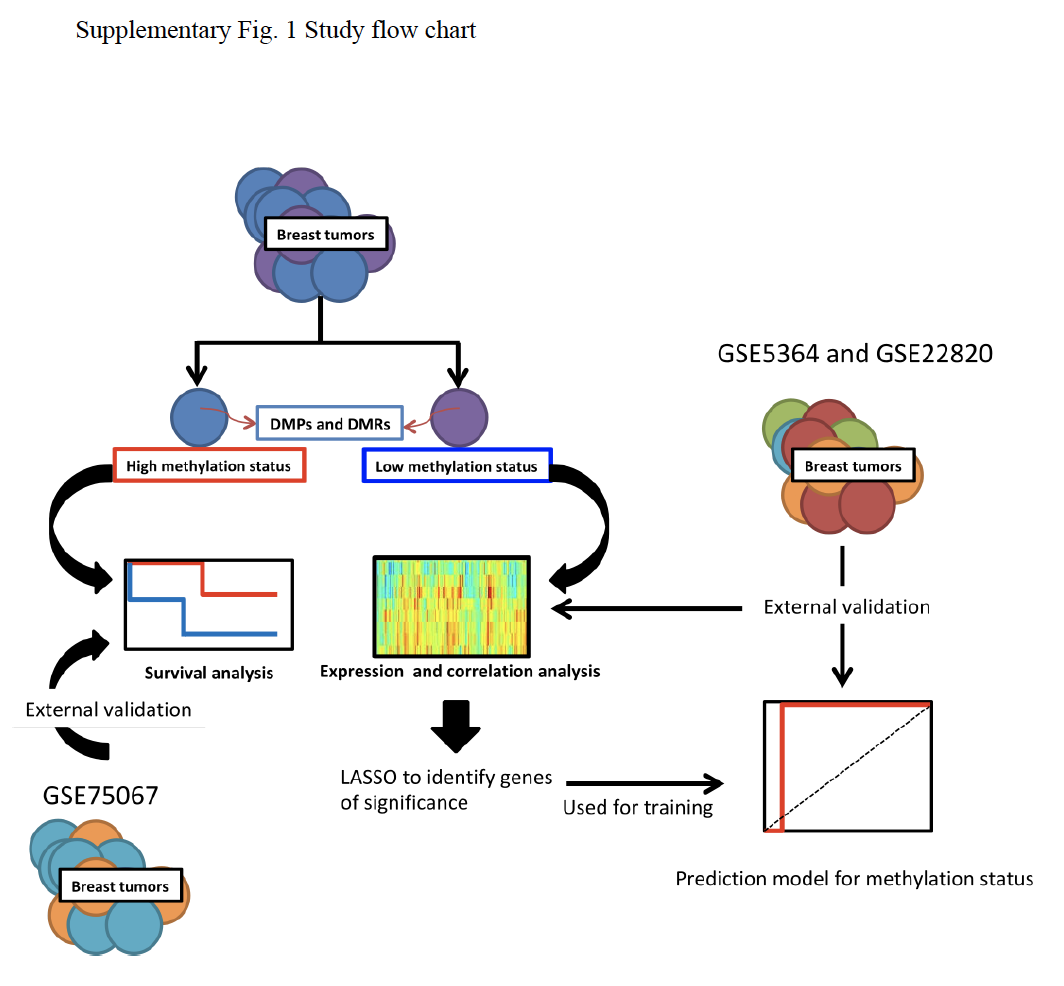

Supplement: Supplementary file 1 — Additional file 1: Figure S1. Study flow chart. [file 12885_2021_8645_MOESM1_ESM.png]

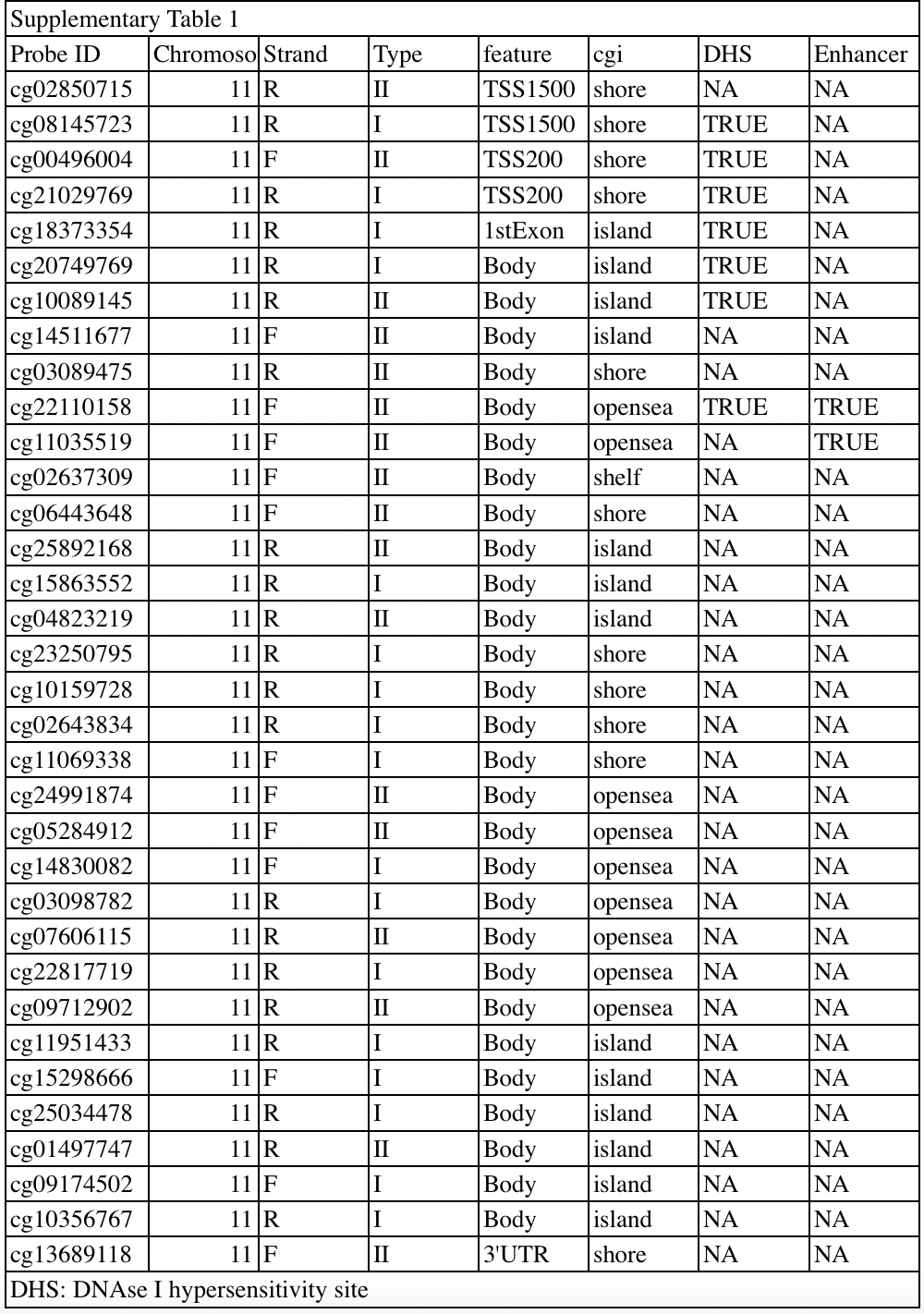

Supplement: Supplementary file 2 — Additional file 2: Table S1. Detailed information of 34 probes annotated with ST14. [file 12885_2021_8645_MOESM2_ESM.png]

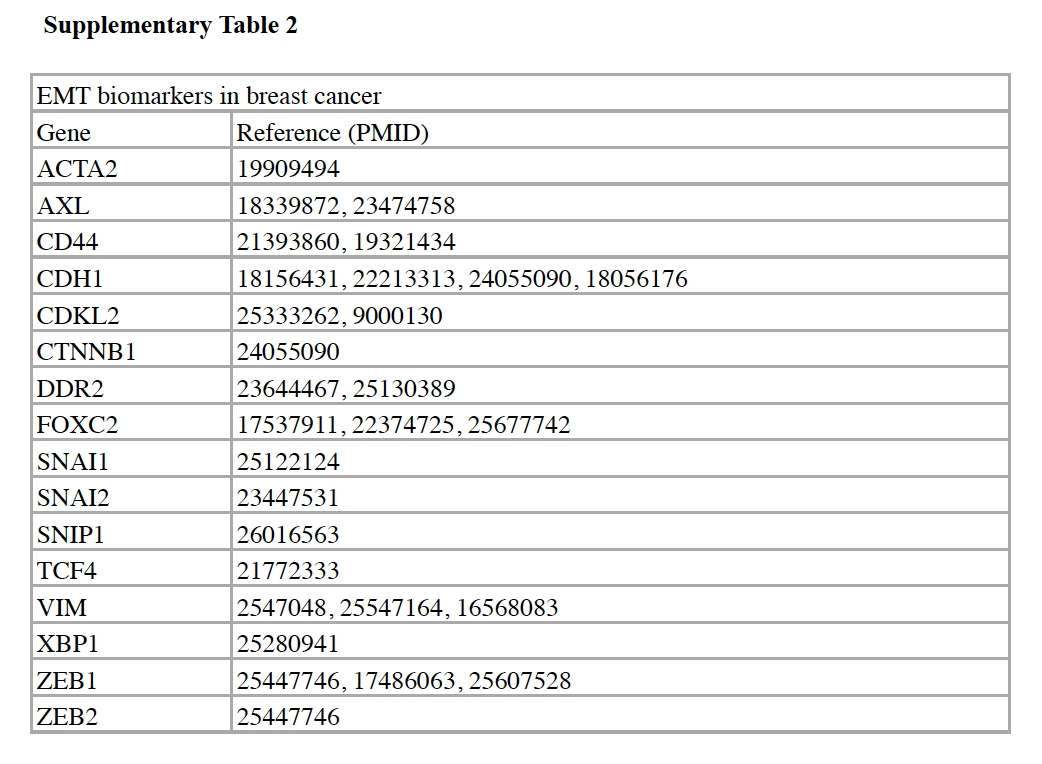

Supplement: Supplementary file 3 — Additional file 3: Table S2. EMT biomarkers in breast cancer. [file 12885_2021_8645_MOESM3_ESM.png]

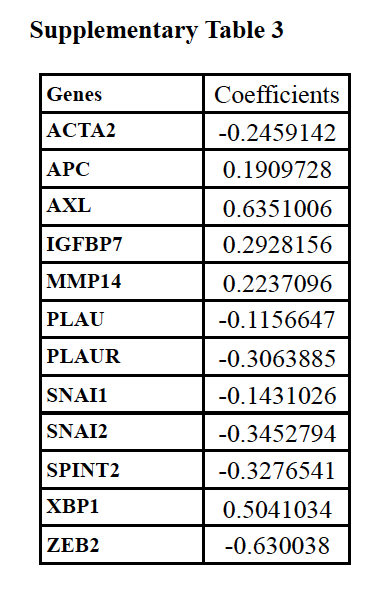

Supplement: Supplementary file 4 — Additional file 4: Table S3. Coefficient of genes derived from LASSO. [file 12885_2021_8645_MOESM4_ESM.png]

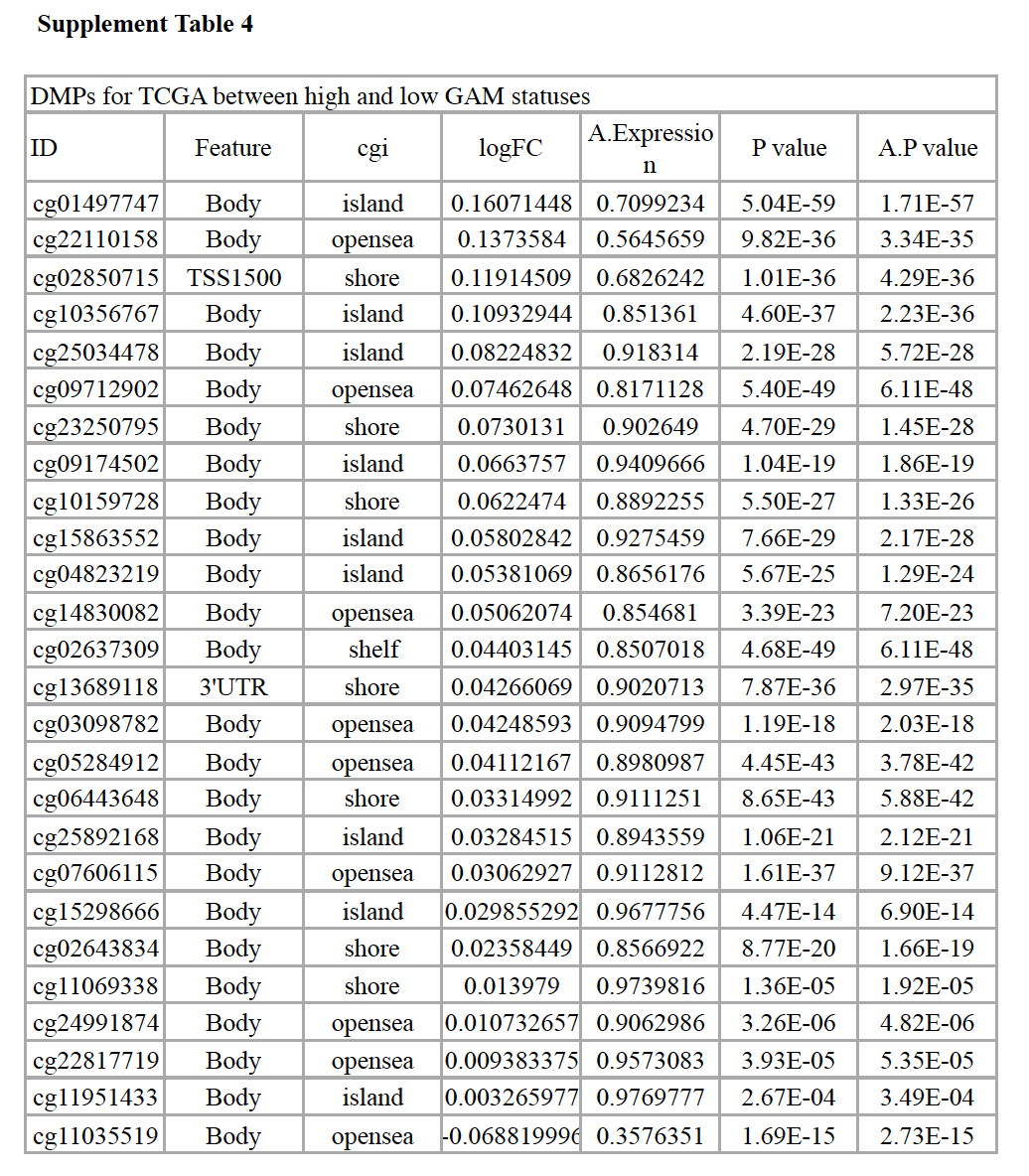

Supplement: Supplementary file 5 — Additional file 5: Table S4. DMPs for TCGA between high and low GAM groups. [file 12885_2021_8645_MOESM5_ESM.png]

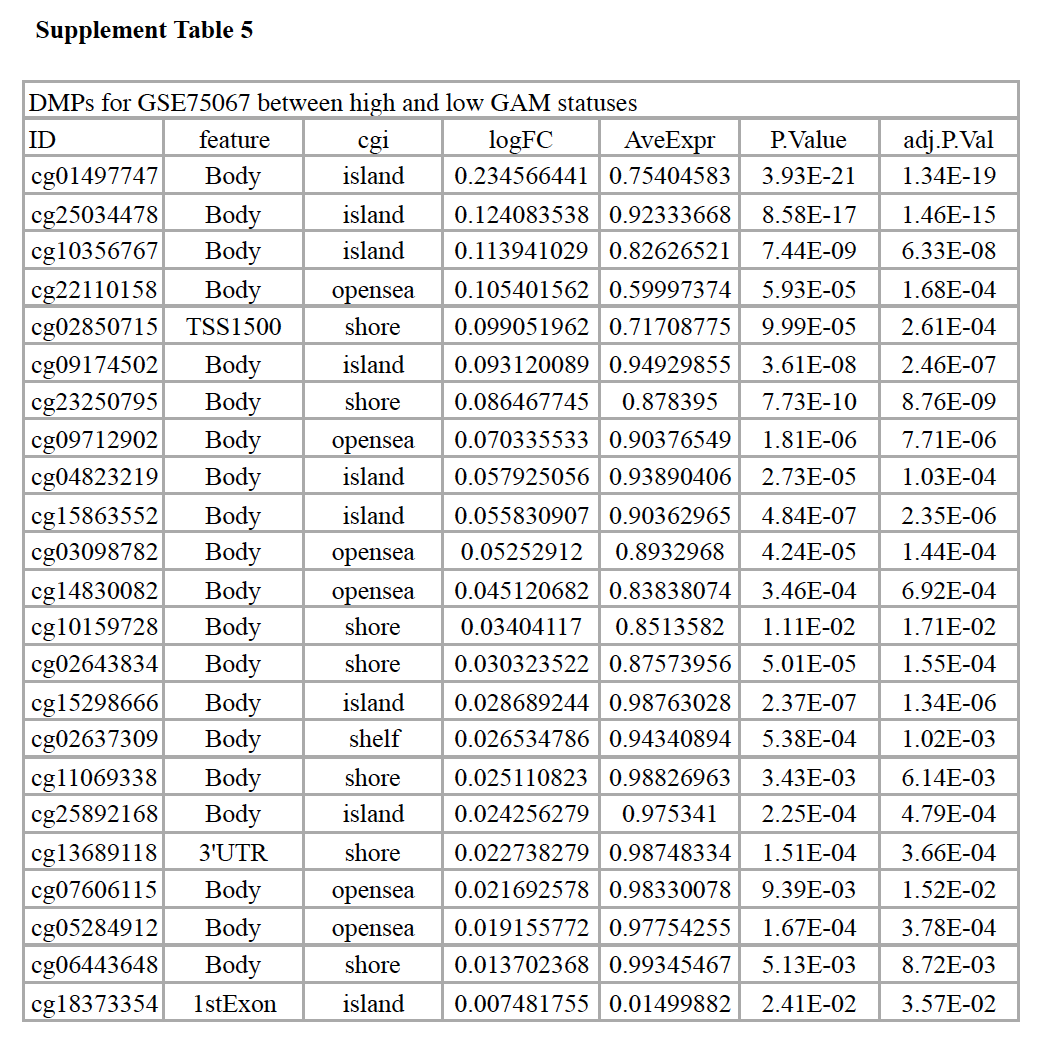

Supplement: Supplementary file 6 — Additional file 6: Table S5. DMPs for GSE75067 between high and low GAM groups. [file 12885_2021_8645_MOESM6_ESM.png]

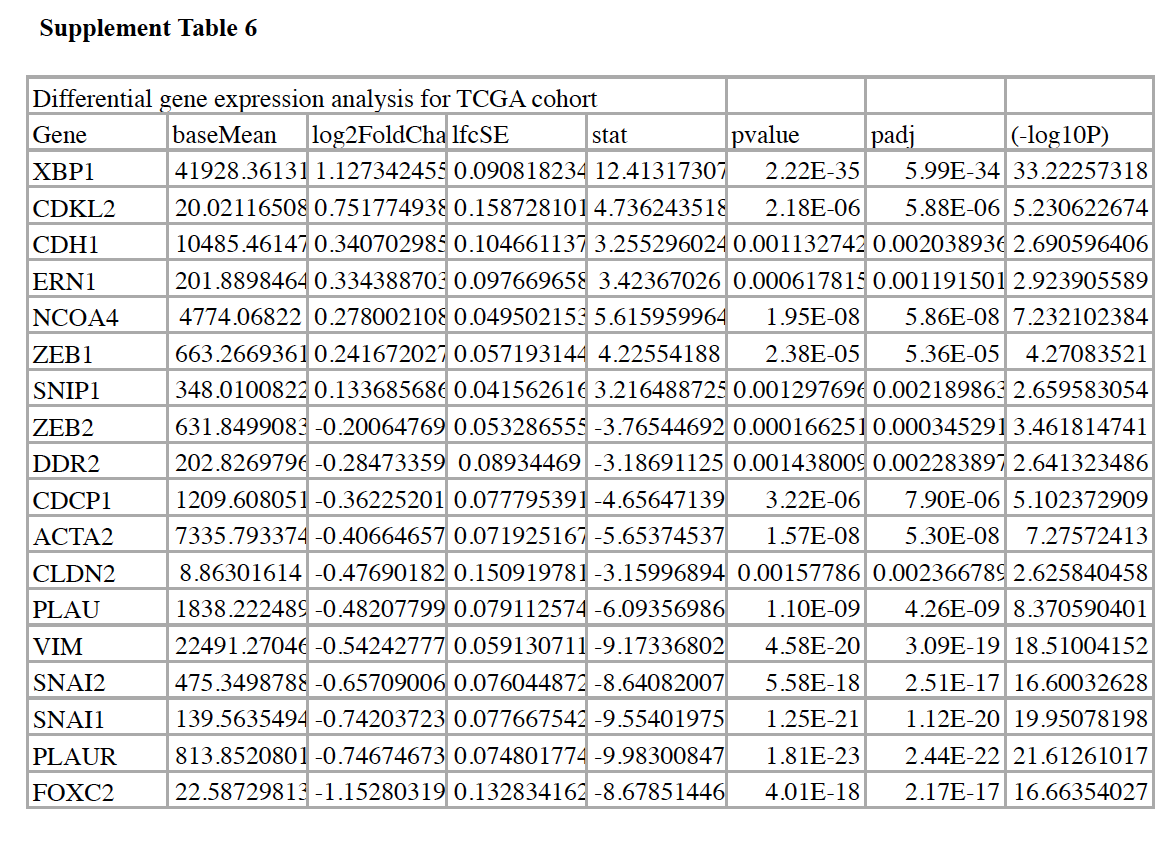

Supplement: Supplementary file 7 — Additional file 7: Table S6. Differential gene expression results for TCGA BRCA cohort. [file 12885_2021_8645_MOESM7_ESM.png]

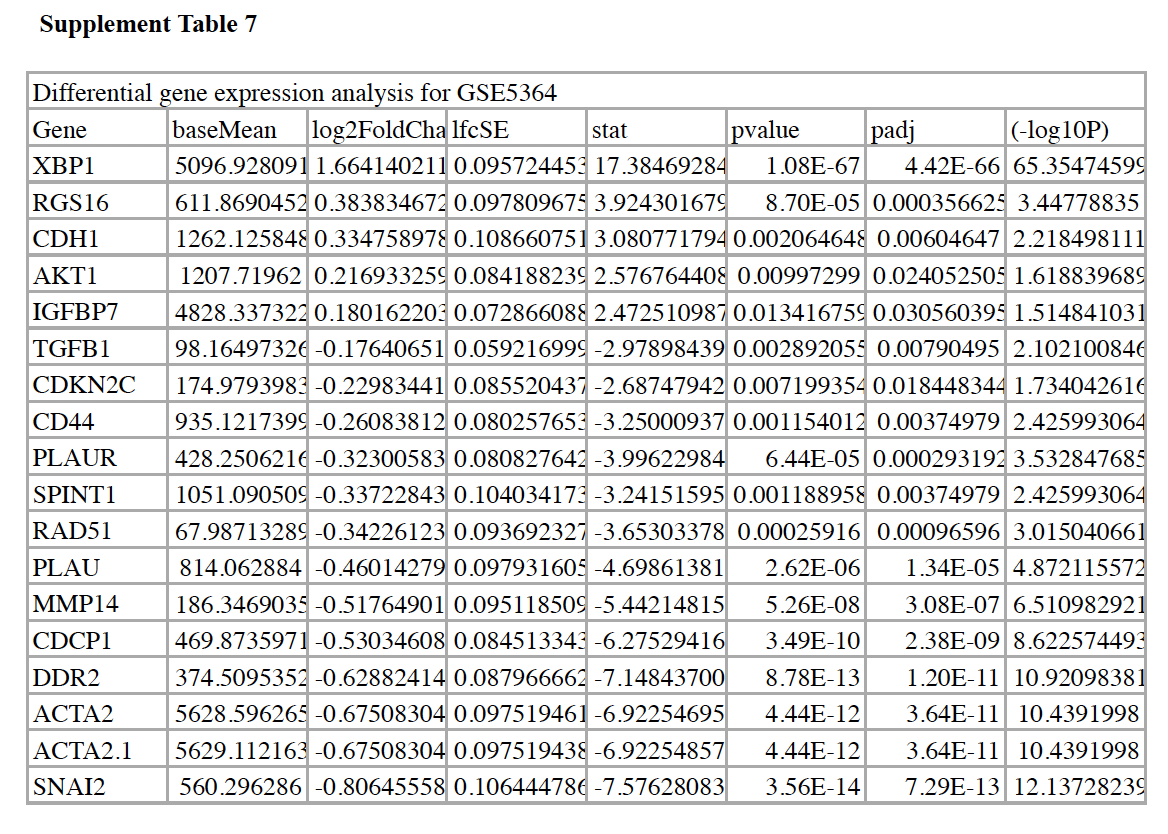

Supplement: Supplementary file 8 — Additional file 8: Table S7. Differential gene expression results for GSE5364. [file 12885_2021_8645_MOESM8_ESM.png]

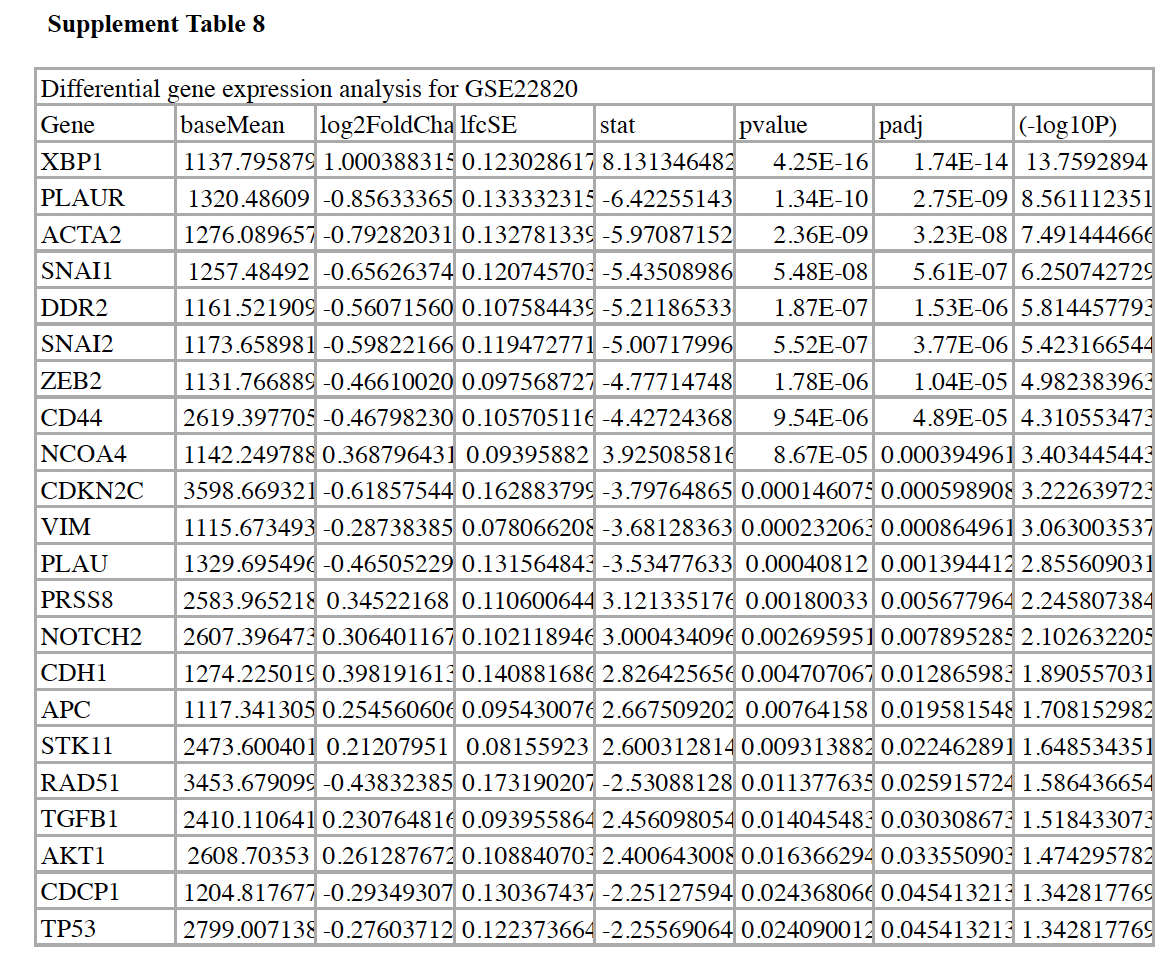

Supplement: Supplementary file 9 — Additional file 9: Table S8. Differential gene expression results for GSE22820. [file 12885_2021_8645_MOESM9_ESM.png]

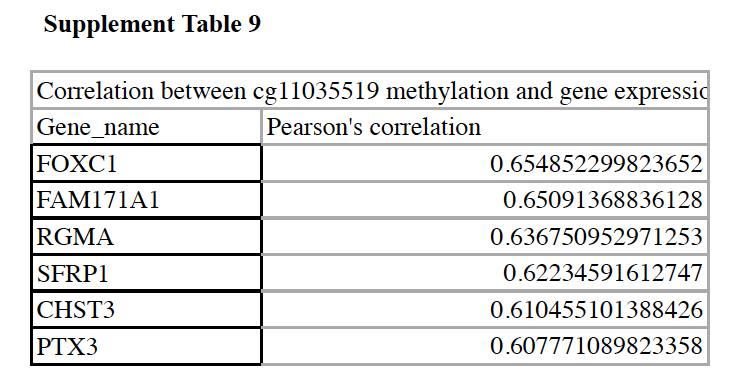

Supplement: Supplementary file 10 — Additional file 10: Table S9. Correlation between cg11035519 and gene expression of FOXC1, FAM171A, RGMA, SFRP1, CHST3, and PTX3. [file 12885_2021_8645_MOESM10_ESM.png]

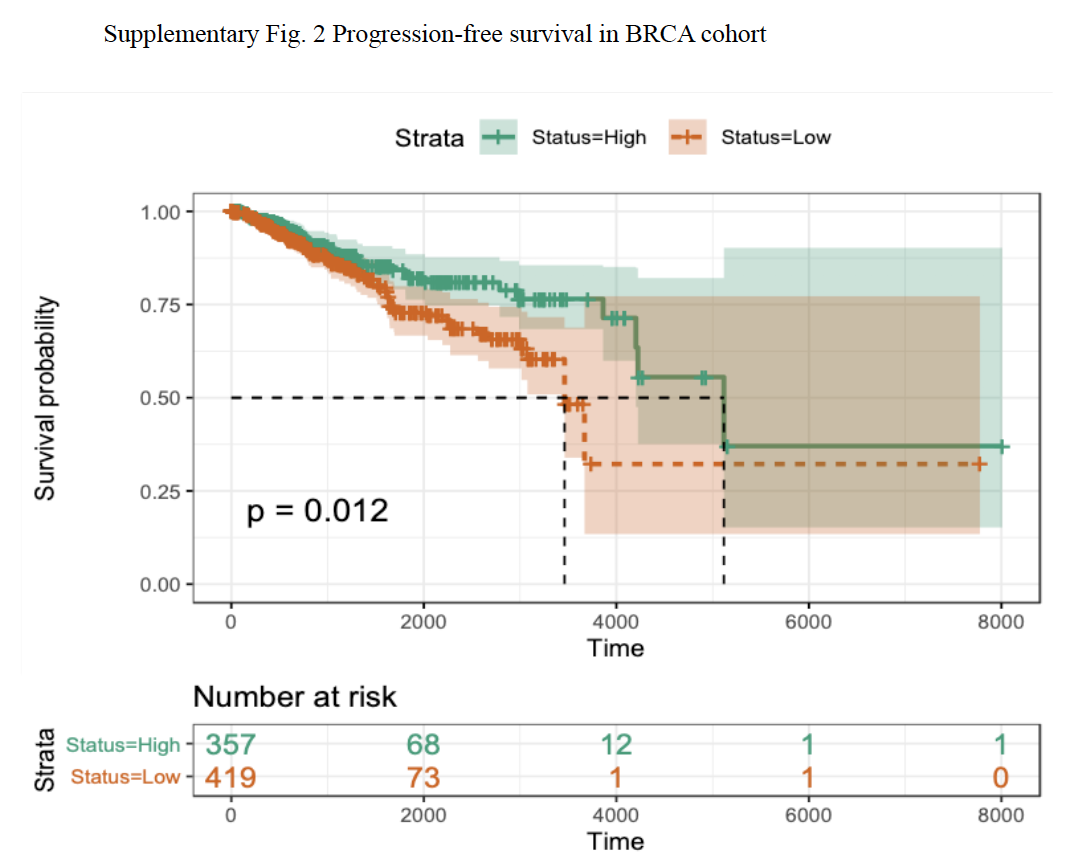

Supplement: Supplementary file 12 — Additional file 12: Figure S2. Progression-free survival in BRCA cohort. [file 12885_2021_8645_MOESM12_ESM.png]

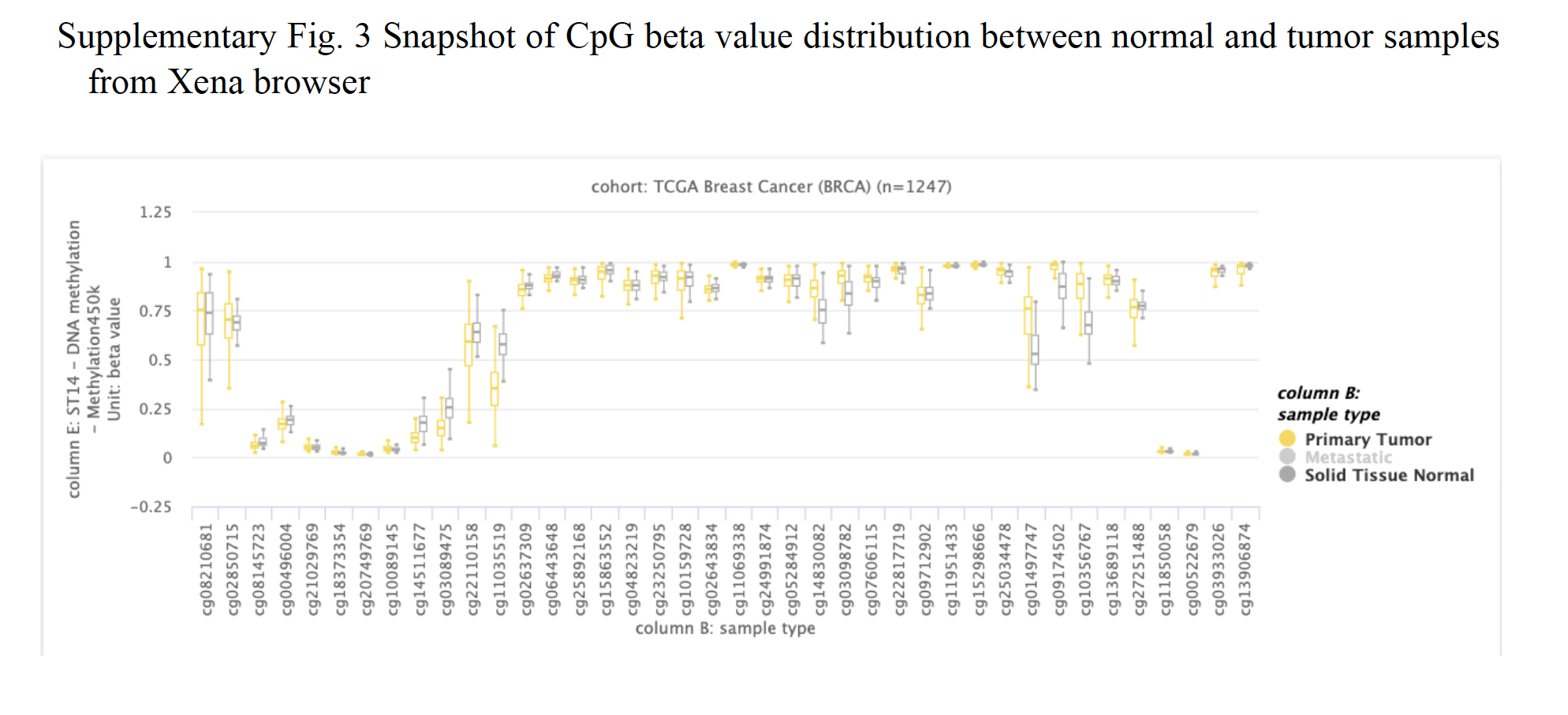

Supplement: Supplementary file 13 — Additional file 13: Figure S3. Snapshot of CpG beta value distribution between normal and tumor samples from Xena browser. [file 12885_2021_8645_MOESM13_ESM.png]

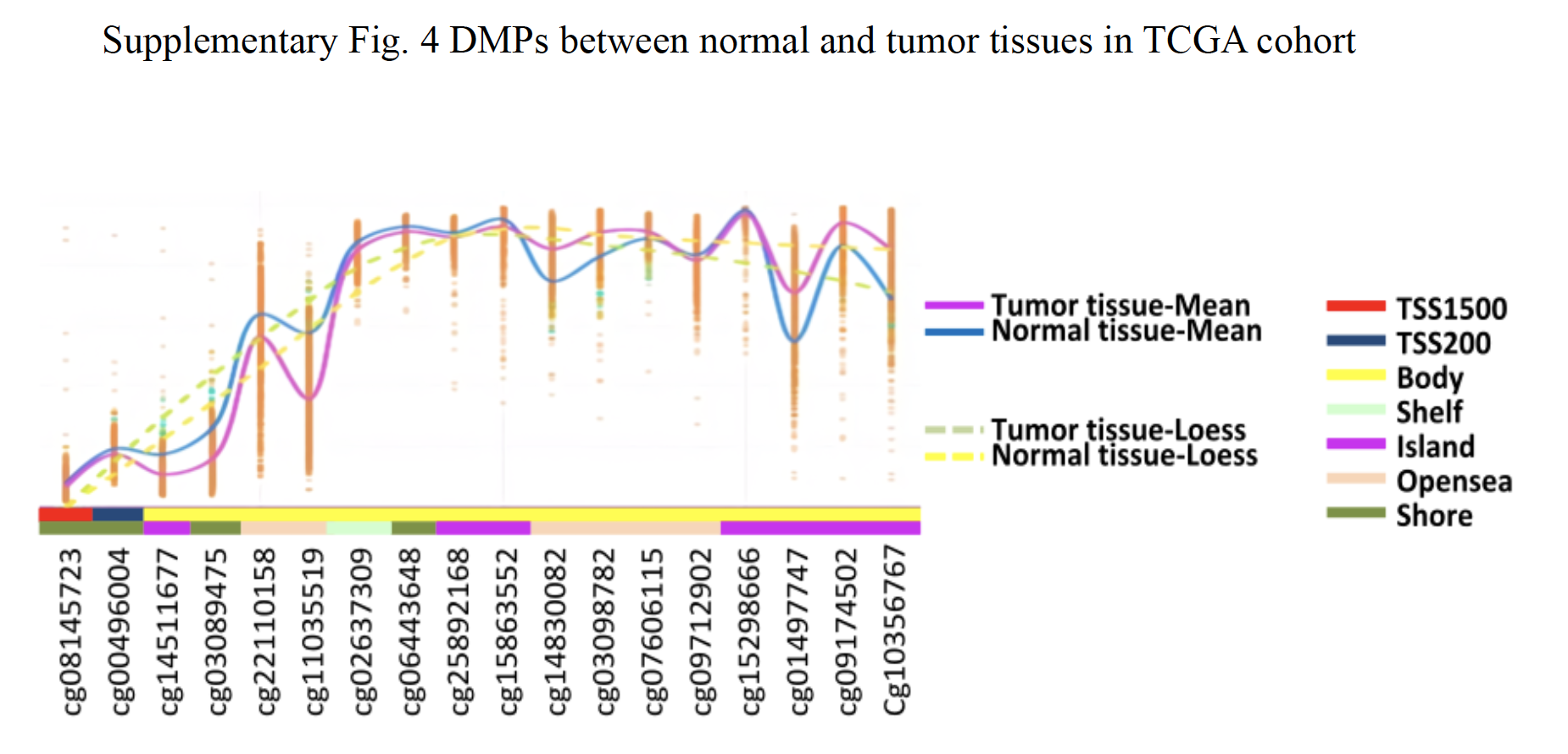

Supplement: Supplementary file 14 — Additional file 14: Figure S4. DMPs between normal and tumor tissues in TCGA BRCA cohort. [file 12885_2021_8645_MOESM14_ESM.png]

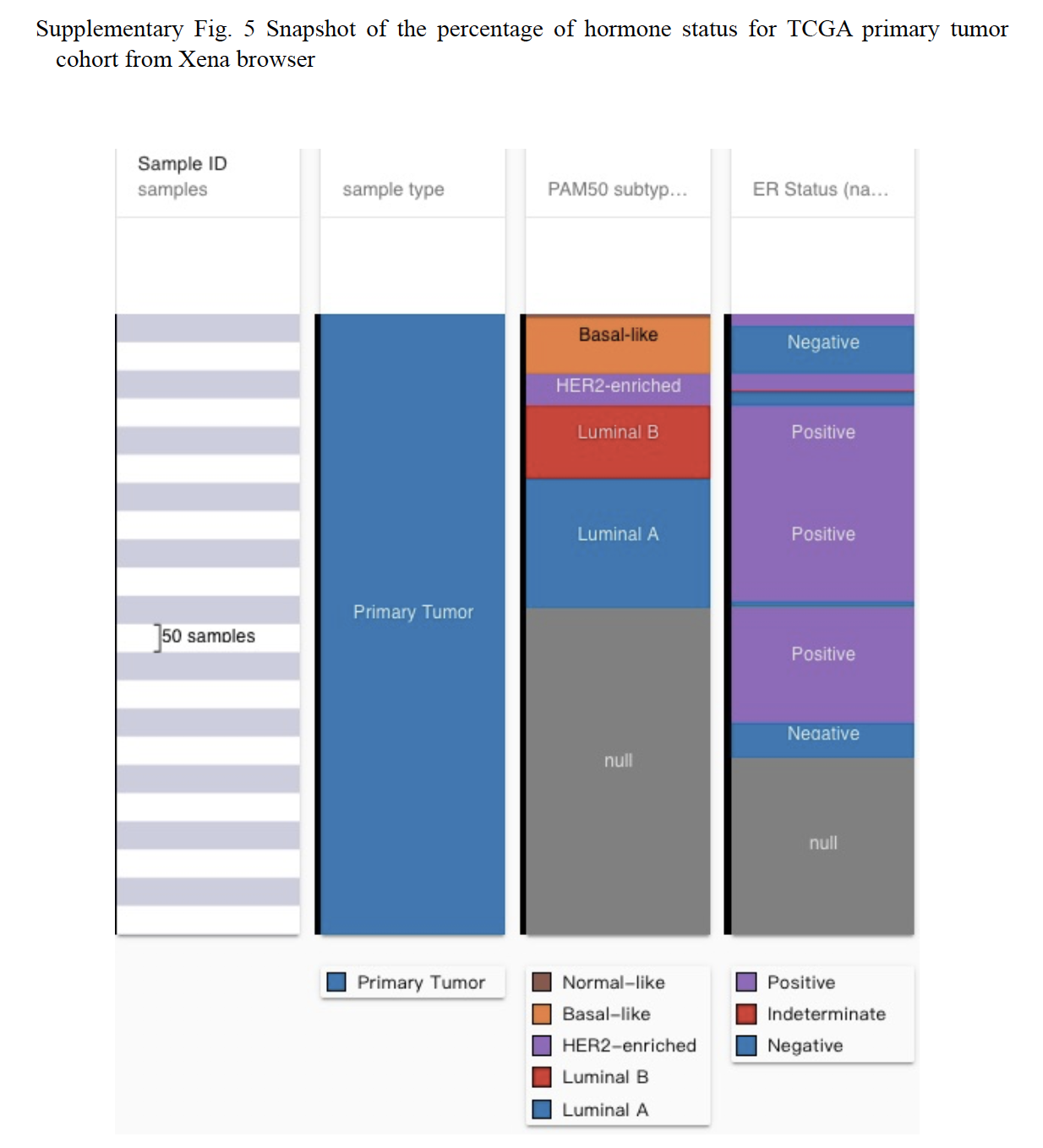

Supplement: Supplementary file 15 — Additional file 15: Figure S5. Snapshot of the percentage of hormone status for TCGA BRCA cohort. [file 12885_2021_8645_MOESM15_ESM.png]
